# Supplementary material for: Evaluation of Virus-Free Chrysanthemum ‘Hangju’ Productivity and Response to Virus Reinfection in the Field: Molecular Insights into Virus–Host Interactions
Source: Plants (Basel). 2024 Mar 5;13(5):732. doi: 10.3390/plants13050732 (PMC10934545; doi:10.3390/plants13050732)
Supplement: Supplementary file 1 [file plants-13-00732-s001.zip › Supplementary_Figure.pptx]

## Slide 1
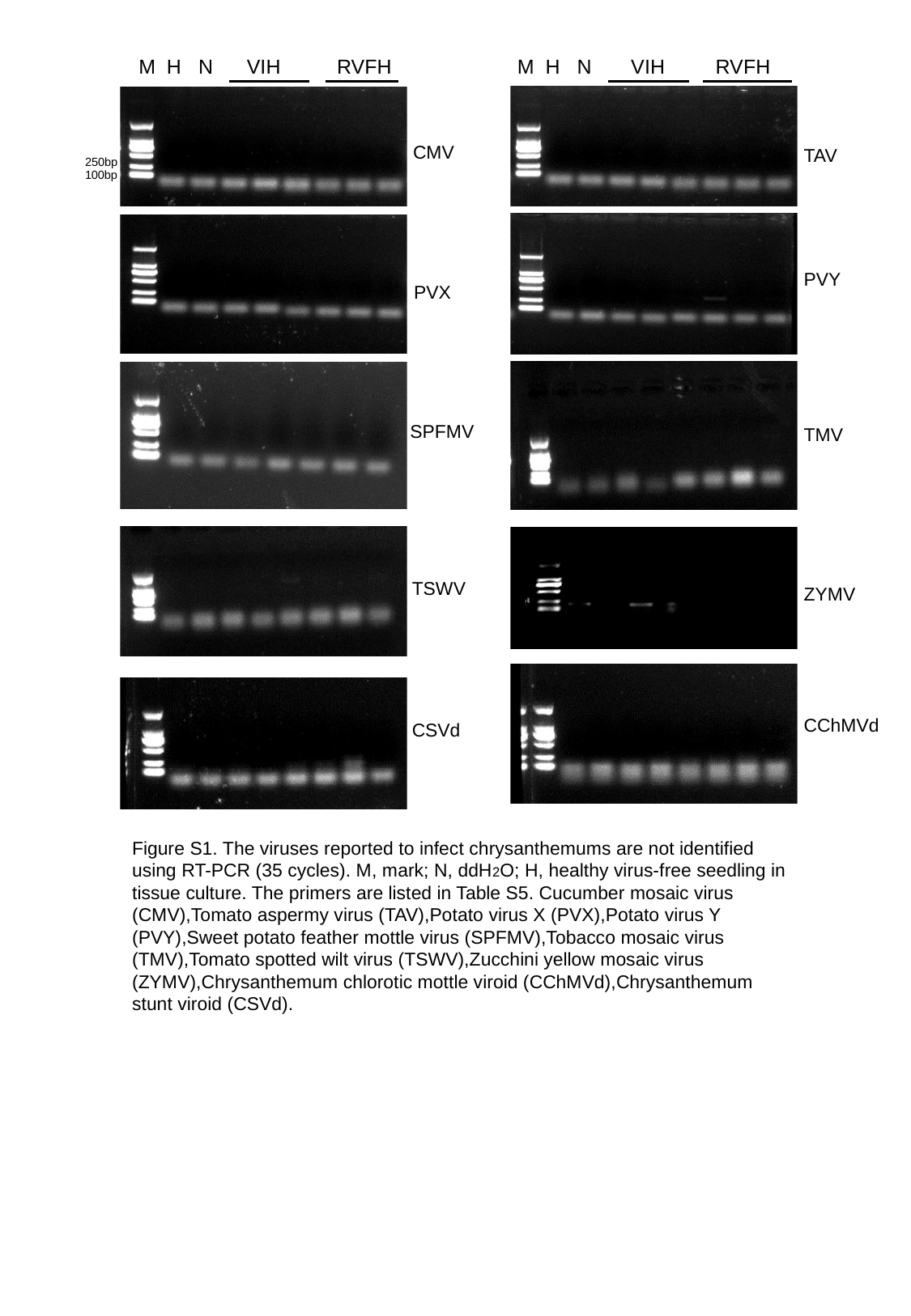

M H N VIH RVFH
M H N VIH RVFH
CMV
TAV
250bp
100bp
PVY
PVX
SPFMV
TMV
TSWV
ZYMV
CChMVd
CSVd
Figure S1. The viruses reported to infect chrysanthemums are not identified using RT-PCR (35 cycles). M, mark; N, ddH2O; H, healthy virus-free seedling in tissue culture. The primers are listed in Table S5. Cucumber mosaic virus (CMV),Tomato aspermy virus (TAV),Potato virus X (PVX),Potato virus Y (PVY),Sweet potato feather mottle virus (SPFMV),Tobacco mosaic virus (TMV),Tomato spotted wilt virus (TSWV),Zucchini yellow mosaic virus (ZYMV),Chrysanthemum chlorotic mottle viroid (CChMVd),Chrysanthemum stunt viroid (CSVd).

## Slide 2
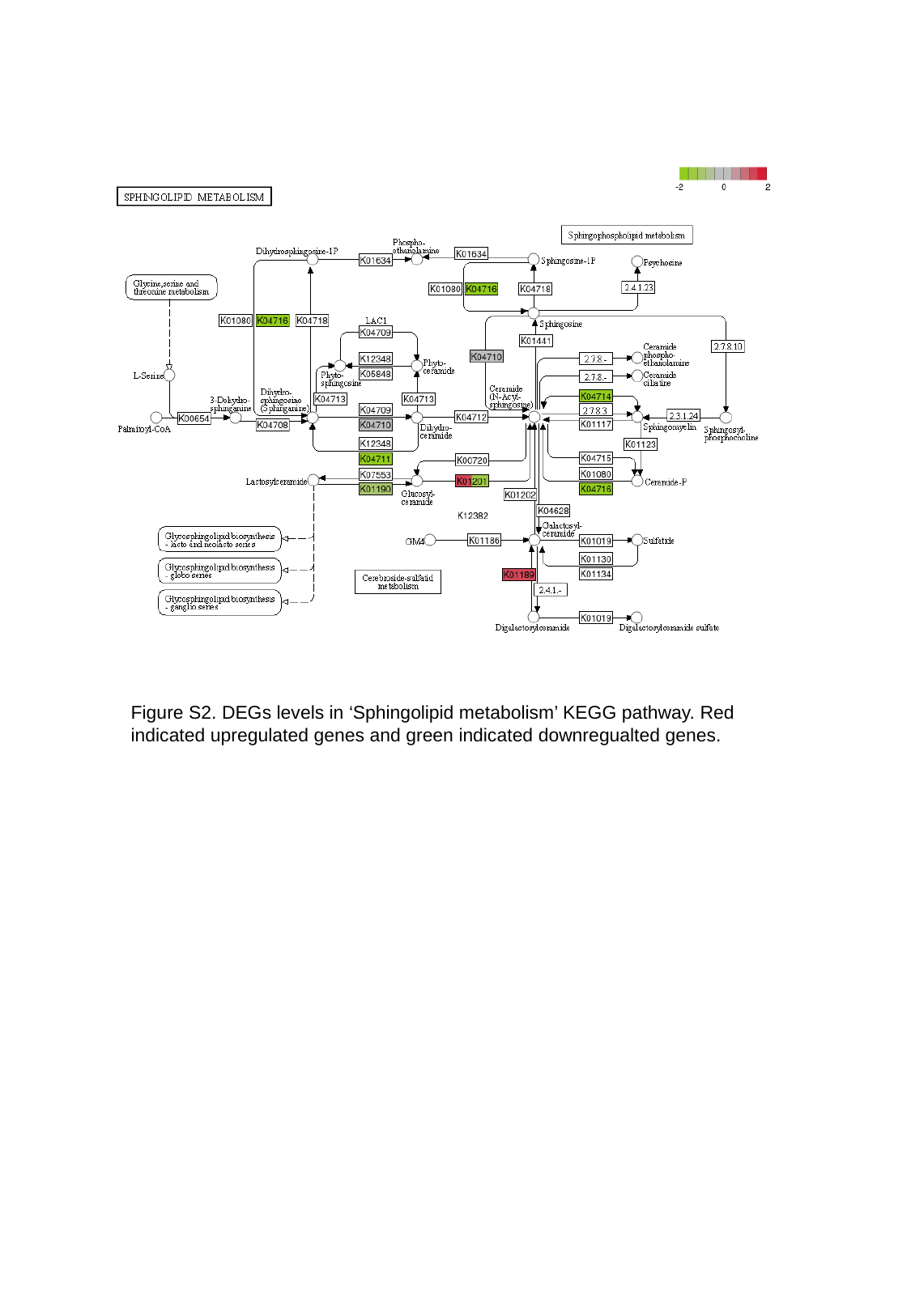

Figure S2. DEGs levels in ‘Sphingolipid metabolism’ KEGG pathway. Red indicated upregulated genes and green indicated downregualted genes.

## Slide 3
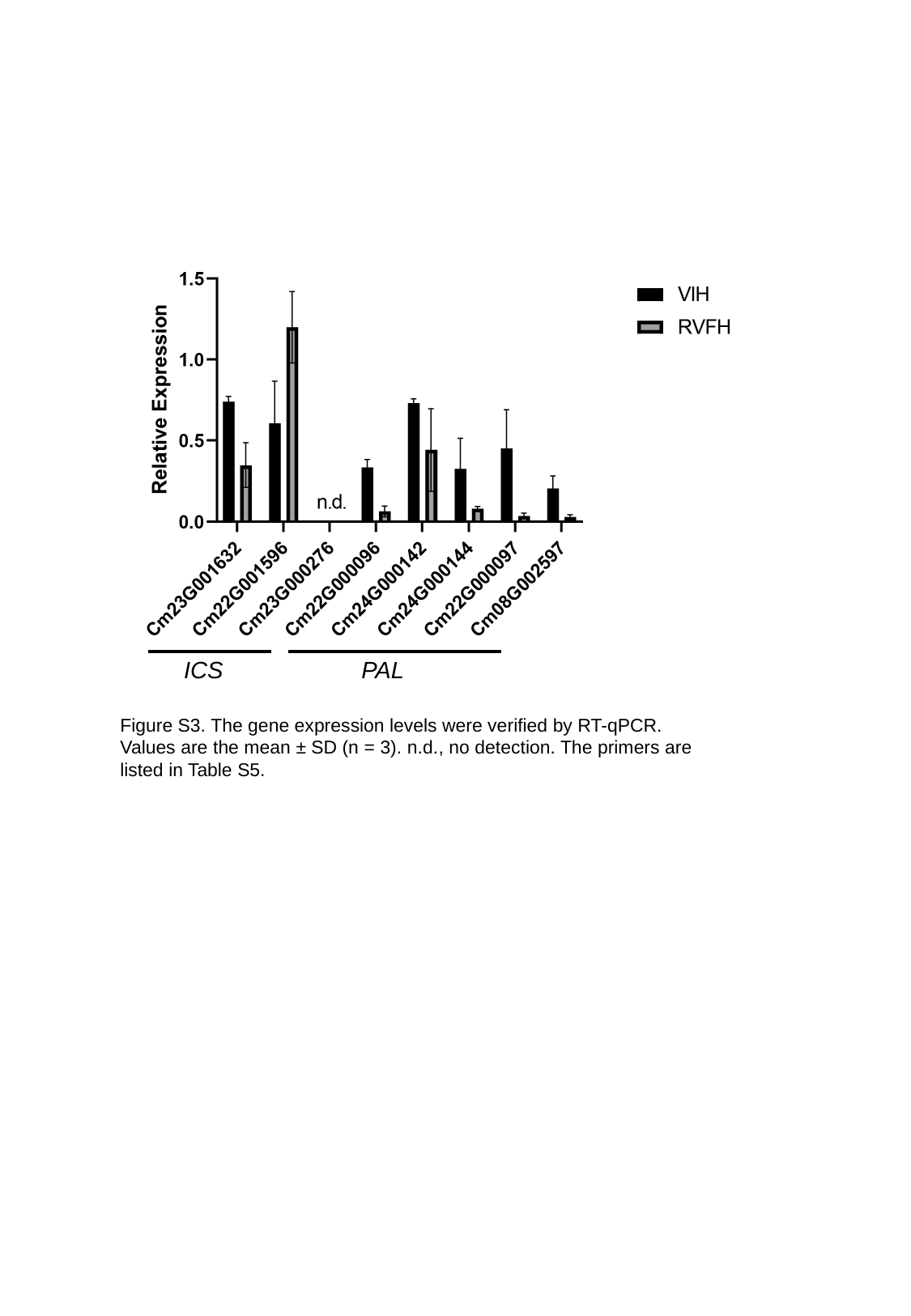

ICS
PAL
Figure S3. The gene expression levels were verified by RT-qPCR. Values are the mean ± SD (n = 3). n.d., no detection. The primers are listed in Table S5.
